# Targeting ectodysplasin promotor by CRISPR/dCas9-effector effectively induces the reprogramming of human bone marrow-derived mesenchymal stem cells into sweat gland-like cells

**DOI:** 10.1186/s13287-017-0758-0

**Published:** 2018-01-12

**Authors:** Sujing Sun, Jun Xiao, Jiahui Huo, Zhijun Geng, Kui Ma, Xiaoyan Sun, Xiaobing Fu

**Affiliations:** 1 Wound Healing and Cell Biology Laboratory, Institute of Basic Medicine Science, College of Life Science, Chinese PLA General Hospital, 28 Fuxing Road, Beijing, 100853 People’s Republic of China; 2 Key Research Laboratory of Tissue Repair and Regeneration of PLA, and Beijing Key Research Laboratory of Skin Injury, Repair and Regeneration, First Affiliated Hospital to the Chinese PLA General Hospital, 51 Fucheng Road, Beijing, 100048 People’s Republic of China; 3 Department of Blood Transfusion, General Hospital of Air Force, PLA, 30 Fucheng Road, Beijing, 100142 People’s Republic of China

**Keywords:** Ectodysplasin (EDA), dCas9-effector, Gene editing, Regeneration, Sweat gland cells

## Abstract

**Background:**

Patients with a deep burn injury are characterized by losing the function of perspiration and being unable to regenerate the sweat glands. Because of their easy accession, multipotency, and lower immunogenicity, bone marrow-derived mesenchymal stem cells (BM-MSCs) represent as an ideal biological source for cell therapy. The aim of this study was to identify whether targeting the promotor of ectodysplasin (EDA) by CRISPR/dCas9-effector (dCas9-E) could induce the BM-MSCs to differentiate into sweat gland-like cells (SGCs).

**Methods:**

Activation of EDA transcription in BM-MSCs was attained by transfection of naive BM-MSCs with the lenti-CRISPR/dCas9-effector and single-guide RNAs (sgRNAs). The impact of dCas9-E BM-MSCs on the formation of SGCs and repair of burn injury was identified and evaluated both in vitro and in a mouse model.

**Results:**

After transfection with sgRNA-guided dCas9-E, the BM-MSCs acquired significantly higher transcription and expression of EDA by doxycycline (Dox) induction. Intriguingly, the specific markers (CEA, CK7, CK14, and CK19) of sweat glands were also positive in the transfected BM-MSCs, suggesting that EDA plays a critical role in promoting BM-MSC differentiation into sweat glands. Furthermore, when the dCas9-E BM-MSCs with Dox induction were implanted into a wound in a laboratory animal model, iodine-starch perspiration tests revealed that the treated paws were positive for perspiration, while the paws treated with saline showed a negative manifestation. For the regulatory mechanism, the expression of downstream genes of NF-κB (Shh and cyclin D1) was also enhanced accordingly.

**Conclusions:**

These results suggest that EDA is a pivotal factor for sweat gland regeneration from BM-MSCs and may also offer a new approach for destroyed sweat glands and extensive deep burns.

**Electronic supplementary material:**

The online version of this article (doi:10.1186/s13287-017-0758-0) contains supplementary material, which is available to authorized users.

## Background

There are millions of patients who suffer the aftereffects of a burn injury every year. Sweat glands are important skin appendages that participate in the regulation of body temperature via an excretory function. Under normal conditions, approximately 25% of heat is eliminated by vaporization of sweat excreted from sweat glands [1]. In the scar tissue of full-thickness burn injury patients, sweat glands cannot undergo a regeneration process. Thus, burn survivors face a long-term problem of secretory function loss and persistent pain. Furthermore, loss of sweat glands leads to the impairment of temperature adjustment which has great influence on life quality for the patients [2]. Therefore, promoting sweat gland transplantation and accelerating the regeneration of sweat glands may be a potential treatment strategy for the patient with a massive deep burn.

Mesenchymal stem cells derived from human bone marrow (BM-MSCs) have been used in stem cell therapy and regeneration medicine to aid wound healing and skin appendage repair [1, 3, 4]. It has been confirmed that BM-MSCs offer a unique avenue for differentiation into sweat gland-like cells (SGCs) in vitro and can develop secretory function after transplantation in vivo [3, 5, 6]. However, it takes a long time using conventional induction methods to induce MSCs into SGCs, and the differentiation ratio cannot be guaranteed, which affects their clinical application.

Recently, a RNA-guided precise DNA cleavage technology—Clustered Regularly Interspaced Short Palindromic Repeats (CRISPR)/CRISPR-associated system (Cas)—has been widely used in genome editing [7–11], increasing the capacity for stem cell-directed differentiation. Among these, CRISPR/Cas9 provides an accurate and flexible technology for stem cell reprogramming [9]. Most notably, Kearns et al. have developed a doxycycline (Dox)-induced inactive DNA nuclease Cas9-effector (dCas9-E) system to address the differentiation status of stem cells [7]. This dCas9-E system consists of the dCas9 fused to a VP16 tetramer activation domain (VP64) under the control of doxycycline (Dox). Single-guide RNAs (sgRNAs) are then designed to hybridize the target sequence. Finally, these combined elements can generate a DNA complex that recognizes a target locus and activates a specific gene.

Several growth factors have been found to be involved in the development of skin and sweat glands (SGs), such as fibroblast growth factor (FGF) [12], epidermal growth factor (EGF) [13], and anhidrotic ectodysplasias [14, 15]. Among these factors, ectodysplasin (EDA) is considered the most important for the development of sweat gland [14, 16]. The EDA gene, which belongs to the tumor necrosis factor (TNF) family, has been confirmed to be crucial in skin appendage formation, especially for sweat glands [17]. EDA mainly regulates sweat gland maturation through activating nuclear factor-κB (NF-κB) after combination with the EDA receptor (EDAR) in the early stage of embryonic development [18]. Thus, the EDA gene could be a therapeutic target for MSC reprogramming into SGCs.

In this study, we choose EDA as the key factor which can trigger a cascade reaction in BM-MSC differentiation into sweat gland-like cells in vitro. sgRNA was designed to combine the upstream EDA gene promoter and lentiviral delivery-based systems with a dCas9-E nuclease or with the sgRNAs, and transfected into BM-MSCs. We hypothesized that EDA is a potential factor in BM-MSC differentiation into sweat gland-like cells, and these reprogrammed cells could be applied for the reconstruction of sweat glands and skin wounds.

## Methods

### Animals

Athymic BALB/c nude mice (male, 6–8 weeks old, 20–25 g body weight) were purchased from Vital River (Beijing, China) and housed under pathogen-free conditions. The animal study was performed according to the protocols approved by the Ethics Committee at the General Hospital of the People’s Liberation Army and carried out in accordance with Institutional Animal Care and Use Committee (IACUC) guidelines.

### Cell culture, chemical reagents and antibodies

Human bone marrow-derived mesenchymal stem cells (BM-MSCs) were purchased from Cyagen Biosciences (HUXMA-90011, Santa Clara, CA, USA) and were cultured in Dulbecco’s modified Eagle’s Medium (DMEM; Gibco, Grand Island, USA) with low glucose in the presence of 10% fetal bovine serum (FBS; Hyclone, USA). MGC-803 cells were maintained in RPMI 1640 supplemented with 10% FBS. All the cells were incubated in a cell culture incubator at 37 °C in a humidified atmosphere containing 5% CO_2_.

Doxycycline (Dox), puromycin, and G418 were purchased from Sigma Life Science (St. Louis, MO, USA). The rabbit monoclonal antibodies used in this study, including carcinoembryonic antigen (CEA), cytokeratin (CK)7, CK14, CK19, Sonic Hedgehog (Shh), and cyclin D1, and rabbit polyclonal antibody to EDA were purchased from Abcam (Cambridge, MA, USA). The hemagglutinin (HA) mouse monoclonal antibody was supplied by CWBio (Beijing, China).

### Single-guide RNA design and lentiviral vector production

**Fig. 1:**
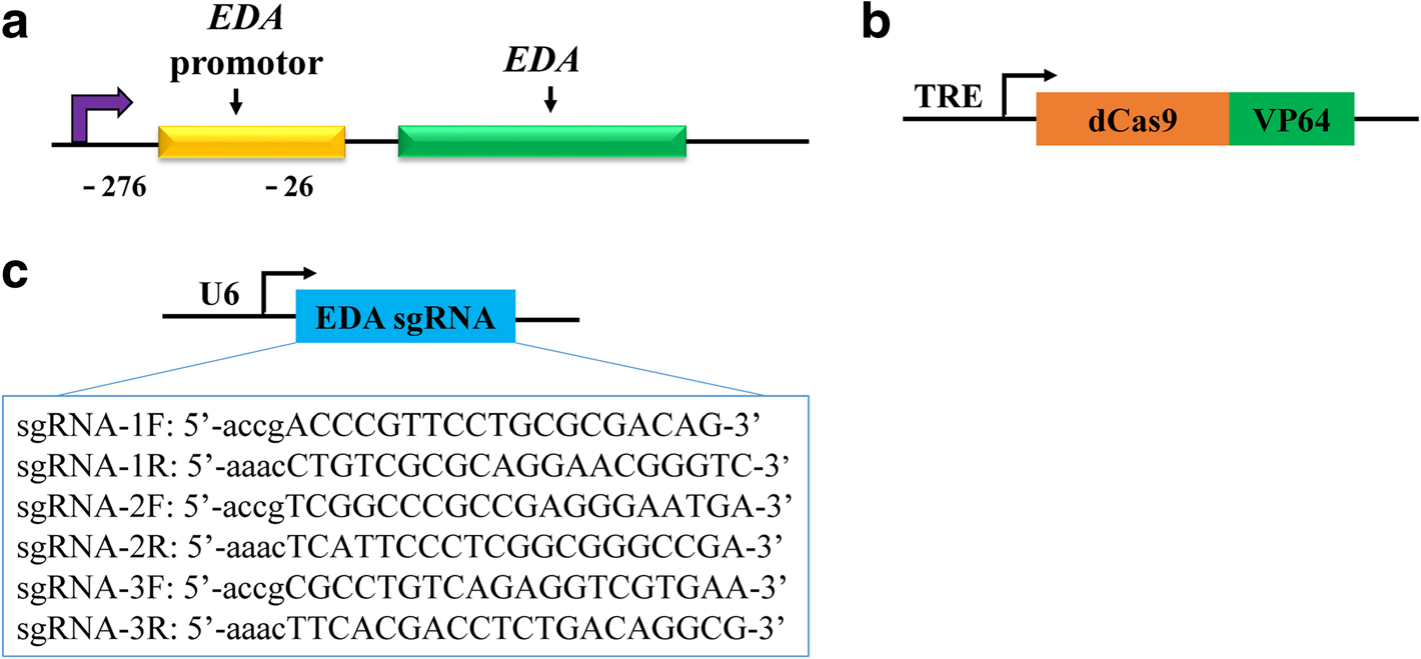
Design of CRISPR/dCas9-E nucleases specific for targeting the ectodysplasin (EDA) promotor. **a** Schematic representation of the EDA genome and **b** scheme of lentiCRISPR/dCas9-E plasmids. **c** Schematic representation of the pLKO.1-puro-U6 vector. A guide-sequence insertion site existed downstream of the U6 promoter for cloning the designed single-guide RNA (sgRNA) by the *BfuA*I restriction site

Candidate sgRNAs were identified by searching for 5’-N_20_GG motifs, 276 bases to 26 bases upstream of the EDA transcriptional start site (TSS), that conformed with the nucleotide requirements for the spCas9 PAM recognition element (NGG). The sgRNAs were designed using an online CRISPR Design Tool (http://crispr.mit.edu; Fig. 1c).

The sgRNA expression plasmid (pLKO.1-puro U6 sgRNA BfuAI stuffer) and the plasmid encoding dCas9-E (pHAGE-TRE-dCas9-VP64) were previously developed by others [7] and were obtained from Addgene (plasmids #50920 and #50916). The sgRNAs containing the target sequences were cloned into the pLKO.1-puro-U6 plasmid using the *BfuA*I sites as described by Kearns et al. [7]. The insertion was verified by clone sequencing.

### Lentiviral production

HEK-293FT cells were maintained in DMEM supplemented with 10% FBS. HEK-293FT cells were split and plated in 10 cm^2^ culture dishes. On the following day, packaging plasmids and dCas9-E or sgRNA-coding plasmids were co-transfected using Lipofectamine® 3000 (Invitrogen) according to the manufacturer’s instructions.

### Generation of stable dCas9-E cell lines and co-expression with sgRNAs

The transduction and selection strategy were as previously described [7]. Briefly, BM-MSCs were incubated with tetracycline-responsive element (TRE)-regulated dCas9-E lentivirus on low-attachment plates. The transduced cells were treated with 1 mg/ml G418 from 48 h after transduction to select and maintain stable cell lines. For experiments utilizing sgRNAs, the stable dCas9-E cell lines were infected with sgRNA lentiviruses. Forty-eight hours later, the sgRNA transduced cells were treated with 1 μg/ml puromycin to select sgRNA-expressing cells and 2 μg/ml doxycycline (Dox) to induce expression of dCas9-E (day 0).

### Immunofluorescence

Cells were fixed with 4% paraformaldehyde (30 min) and then incubated in 1% bovine serum albumin (BSA) for 1 h. The cells then incubated with primary anti-human antibodies (1:500) overnight at 4 °C. The primary antibodies were decanted and the cells were incubated with phycoerythrin (PE)-conjugated or fluorescein isothiocyanate (FITC)-conjugated goat anti-Rabbit or Mouse IgG secondary antibody (1:1000; Santa Cruz) for 1 h at room temperature in the dark. The cells were finally stained with 4’6-diamidino-2-phenylindole (DAPI; Sigma) and then visualized and examined by a Leica fluorescence microscope.

### Quantitative reverse transcription polymerase chain reaction (qRT-PCR)

RNA was extracted from BM-MSCs and dCas9-E cells using TRIzol reagent (Invitrogen) at 48 h after Dox (2 μg/ml) treatment. Total RNA (1.0 μg) was reverse-transcribed into cDNA using ReverTra Ace qRCR RT Master Mix with gDNA Remover (TOYOBO, Osaka, Japan). Each sample was measured in triplicate. The primer sequences of the genes, including CEA, EDA, CK19, Shh, and cyclin D1, are listed in Additional file 1. All genes were normalized to the endogenous reference gene glyceraldehyde-3-phosphate dehydrogenase (GAPDH). The relative expression levels in cells were calculated as fold changes.

### Western blot

BM-MSCs and dCas9-E BM-MSCs were harvested after being treated with Dox (2 μg/ml) for 48 h. Cells were lysed in a passive lysis buffer (Promega, Madison, WI, USA) with a cocktail of protease inhibitors (Roche, Mannheim, Germany) at 4 °C for 30 min and then centrifuged at 12,000 × g for 10 min at 4 °C. Total protein (30 μg) was separated by 12% SDS-PAGE and transferred to polyvinylidene difluoride (PVDF) membranes (Millipore, Billerica, MA, USA). After blocking with 5% (*w/v*) BSA (MP, Auckland, New Zealand) for 1 h, the membranes were probed with primary antibodies overnight at 4 °C. The membranes were then incubated with horseradish peroxidase (HRP)-conjugated secondary antibodies for 1.5 h. Specific bands were visualized using a luminol reagent (Santa Cruz Biotechnology).

### Transmission electron microscopy (TEM)

After Dox treatment, BM-MSCs and dCas9-E BM-MSCs were harvested and fixed with 2.5% glutaraldehyde in phosphate buffer overnight. Cell samples were first dehydrated by a graded series of ethanol for 15 min at each step and transferred to absolute acetone for 20 min. Resin was then used for sample infiltrating and embedding. Samples were prepared as ultrathin sections and observed with the Hitachi TEM system.

### Animal studies

BM-MSCs transfected with dCas9-E and pLKO.1-sgRNAs were implanted into a scald injury animal model. The transplantation procedure was performed as previously described [15]. Briefly, full thickness scald injuries were made on both paws of the hind legs of 10 athymic BALB/c nude mice. The right scalded paw of each mouse received a subcutaneous injection with 1 × 10^6^ Dox-induced dCas9-E BM-MSCs in 100 μl medium. The contralateral (sham) scalded paw was subcutaneously injected with saline. The injury sites were photographed and samples collected every week to measure wound healing and re-epithelialization in each group. Twenty days later an iodine-starch perspiration test was performed, and skin biopsies were also examined by histology. Hematoxylin and eosin staining was used for evaluating the extent of re-epithelialization. Masson and Sirius red staining were used to observe paw fibrosis after injury.

Tumorigenicity tests were generated by subcutaneous injection of either MGC-803 cells (5 × 10^5^ cells) or Dox-induced dCas9-E BM-MSCs (5 × 10^5^ cells) as previously described [19]. Twelve weeks after cell inoculation, hematoxylin and eosin staining was used to measure tumor formation.

### Statistical analysis

Statistical analyses were performed using SPSS (V.20) statistical software (SPSS Inc., Chicago, IL, USA). All values are expressed as the mean ± standard deviation. Significant differences were calculated by one-way analysis of variance (ANOVA) followed by the Bonferroni test when performing multiple comparisons between groups. A *p* value lower than 0.05 was considered as a statistically significant difference.

## Results

### Design of the EDA-targeting CRISPR/dCas9-E system

**Fig. 2:**
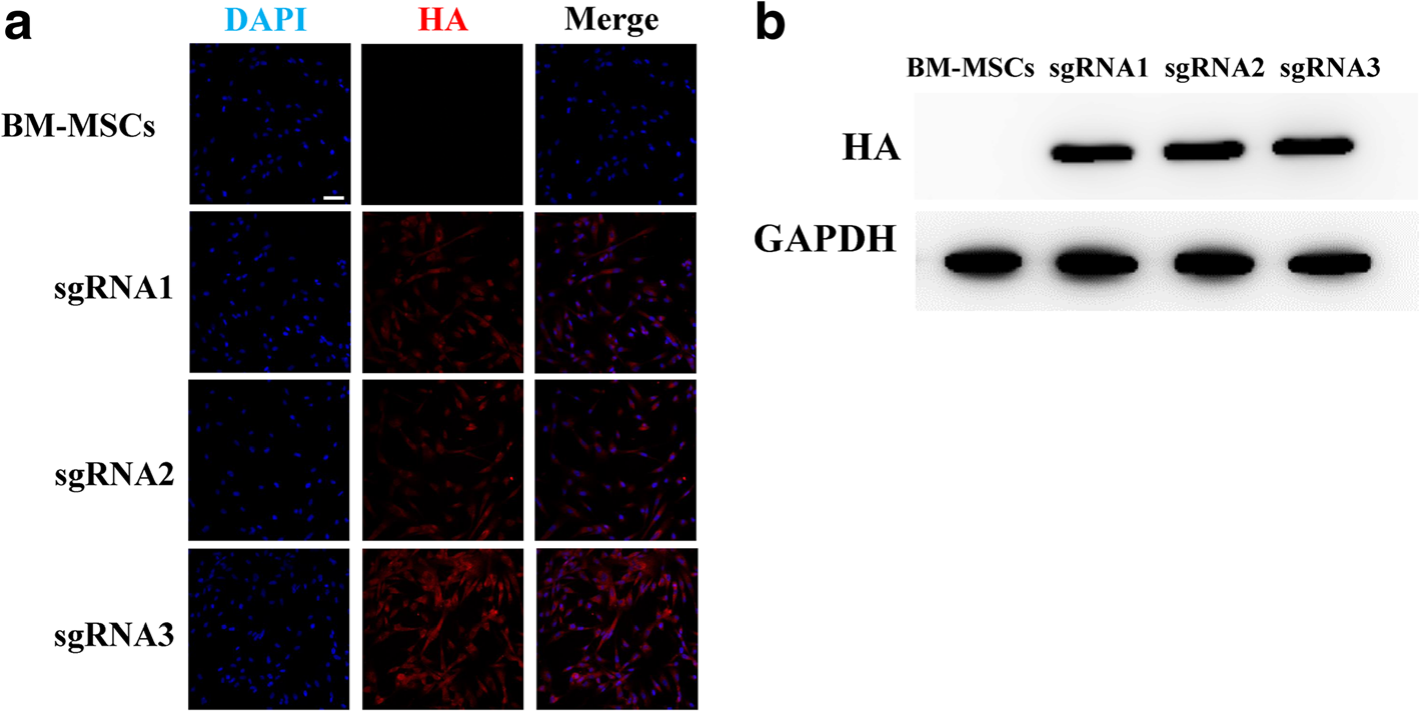
dCas9-E expression in BM-MSCs. **a** Bone marrow-derived mesenchymal stem cells (BM-MSCs) were transfected with pLKO.1-puro-U6 and dCas9-E, and stained with PE-labeled anti-HA and DAPI 72 h post-transfection. **b** The expression of designed dCas9-E nucleases. Scale bar = 50 μm. DAPI 4’6-diamidino-2-phenylindole, GAPDH glyceraldehyde-3-phosphate dehydrogenase, HA hemagglutinin, sgRNA single-guide RNA

The EDA gene, which belongs to the TNF family, has been confirmed to be crucial in sweat gland maturation. Therefore, upregulation of EDA expression may be a feasible way to generate sweat gland cells in vitro. To assess the ability of dCas9-E to upregulate expression of EDA in BM-MSCs, plasmids consisting of a U6 promoter-based lentiviral delivery system for single-guide RNA (sgRNA) to three different target regions upstream of the EDA TSS (Fig. 1a, c) and Dox-inducible expression of dCas9-E under the control of TRE promoters (Fig. 1b) as described by Kearns et al. [7] were obtained from Addgene. An HA marker fused after the dCas9-E protein allowed identification of dCas9-E (Fig. 1b). After identification of the BM-MSCs (Additional file 2), the cells were stable transfected with dCas9-E lentiviral and the HA marker was assessed by immunofluorescence (Fig. 2a) and Western blotting analysis (Fig. 2b).

### The transcription and translation of EDA in dCas9-E BM-MSCs

**Fig. 3:**
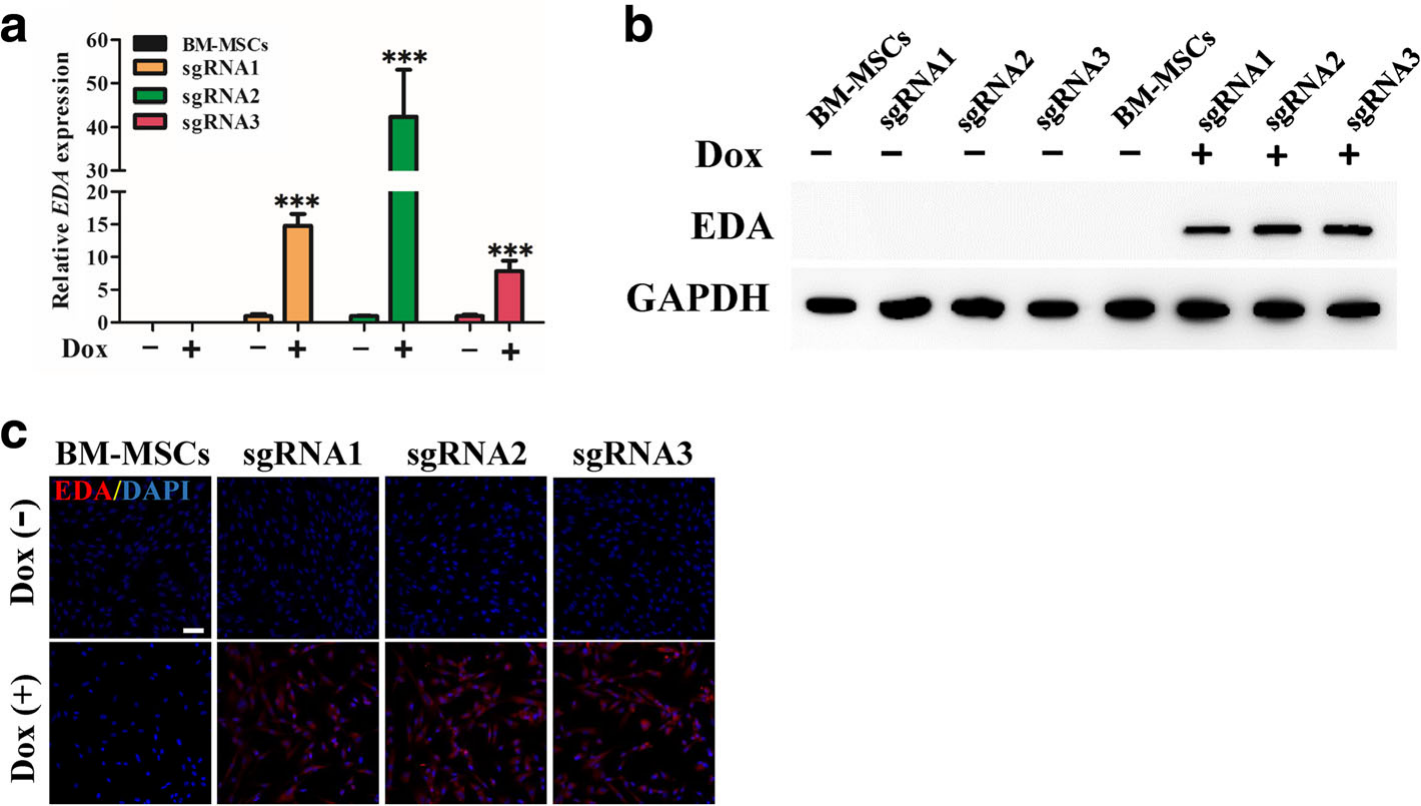
dCas9-E mediated activation of EDA in BM-MSCs. The cells were harvested and total mRNA and protein were extracted. Ectodysplasin (EDA) gene and protein expression were identified by qPCR (**a**) and Western blotting (**b**) after doxycycline (Dox) induction for 48 h. **c** Fluorescence microscopy detection of EDA expression activated by dCas9-E. After supplementing with 2 μg/ml Dox for 48 h, enhanced expression of EDA (red) was detected in dCas9-E transfected bone marrow-derived mesenchymal stem cells (BM-MSCs). The data are expressed as the mean ± SD. ***p < 0.001 for Dox-treated cells versus untreated cells by Dunnett’s test. Scale bar = 50 μm. DAPI 4’6-diamidino-2-phenylindole, GAPDH glyceraldehyde-3-phosphate dehydrogenase, sgRNA single-guide RNA

qRT-PCR analysis showed that the levels of EDA gene transcription were significantly increased in dCas9-E BM-MSCs after Dox induction (Fig. 3a). Consistent with the EDA gene expression levels, the Western blot and immunofluorescence results also indicated that EDA protein expression was enhanced by the dCas9-E, and that the sgRNA2 target to the region (–225/–244) of the EDA TSS was more effective than other two sgRNAs (Fig. 3b, c). Thus, the designed sgRNA2 was used in the subsequent experiments.

### Dox induced dCas9-E BM-MSCs into sweat gland-like cells in vitro

**Fig. 4:**
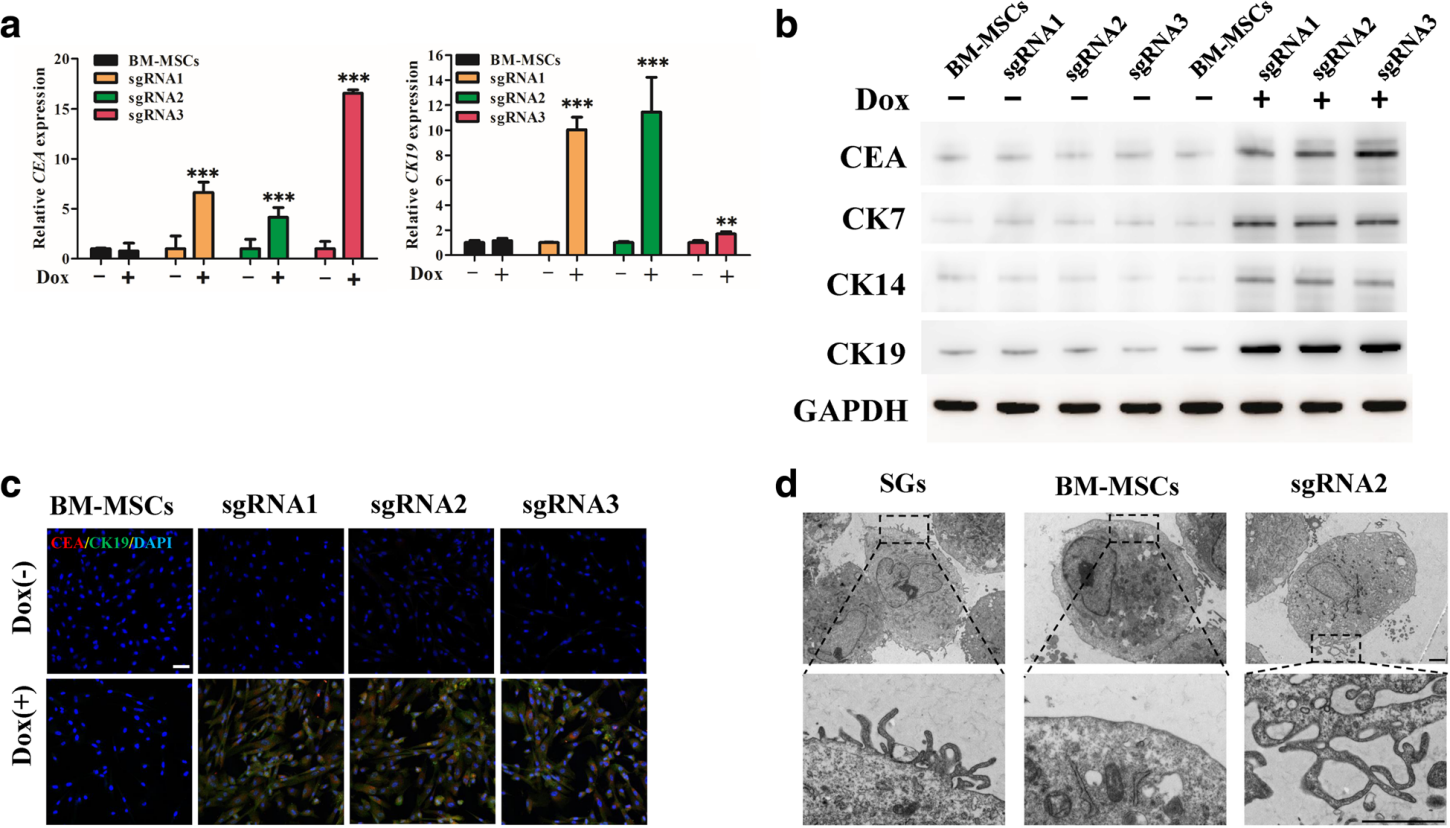
Characteristics of dCas9-E BM-MSCs after Dox induction. **a** The sweat gland markers, carcinoembryonic antigen (CEA) and cytokeratin (CK)19, were detected by qRT-PCR after bone marrow-derived mesenchymal stem cells (BM-MSCs) were transfected with dCas9-E and incubated with doxycycline (Dox; 2 μg/ml) for 48 h. **b** Proteins were collected from BM-MSCs transfected with dCas9-E. The sweat glands biomarkers CEA, CK7, CK14, and CK19 were detected by Western blotting using glyceraldehyde-3-phosphate dehydrogenase (GAPDH) for calibration of sample loading. **c** Immunofluorescence staining was used to detect CEA (red) and CK19 (green) in BM-MSCs (scale bar = 50 μm). **d** Villi ultrastructure changes after dCas9-E BM-MSCs were treated with Dox (scale bar = 1 μm). **p < 0.01, ***p < 0.001 for Dox-treated cells versus untreated cells by Dunnett’s test. DAPI 4’6-diamidino-2-phenylindole, sgRNA single-guide RNA

In order to evaluate the differentiation efficiency of dCas9-E BM-MSCs, cells were analyzed by qRT-PCR and immunofluorescence analysis with the SG biomarkers CEA and CK19, respectively. As expected, dCas9-E BM-MSCs strongly expressed those two SG markers under the induction of Dox; however, BM-MSCs not transfected with dCas9-E were negative (Fig. 4a, c). Furthermore, CEA, CK7, CK14, and CK19 were also detected by Western blotting and those markers were expressed after the induction of Dox (Fig. 4b). TEM examination of cell ultrastructure showed that after Dox induction dCas9-E BM-MSCs had villi structure characteristic of sweat glands (Fig. 4d). These results indicated that dCas9-E BM-MSCs could be induced into SGCs.

### Evaluation of dCas9-E BM-MSCs in a sweat gland injured mouse model

**Fig. 5:**
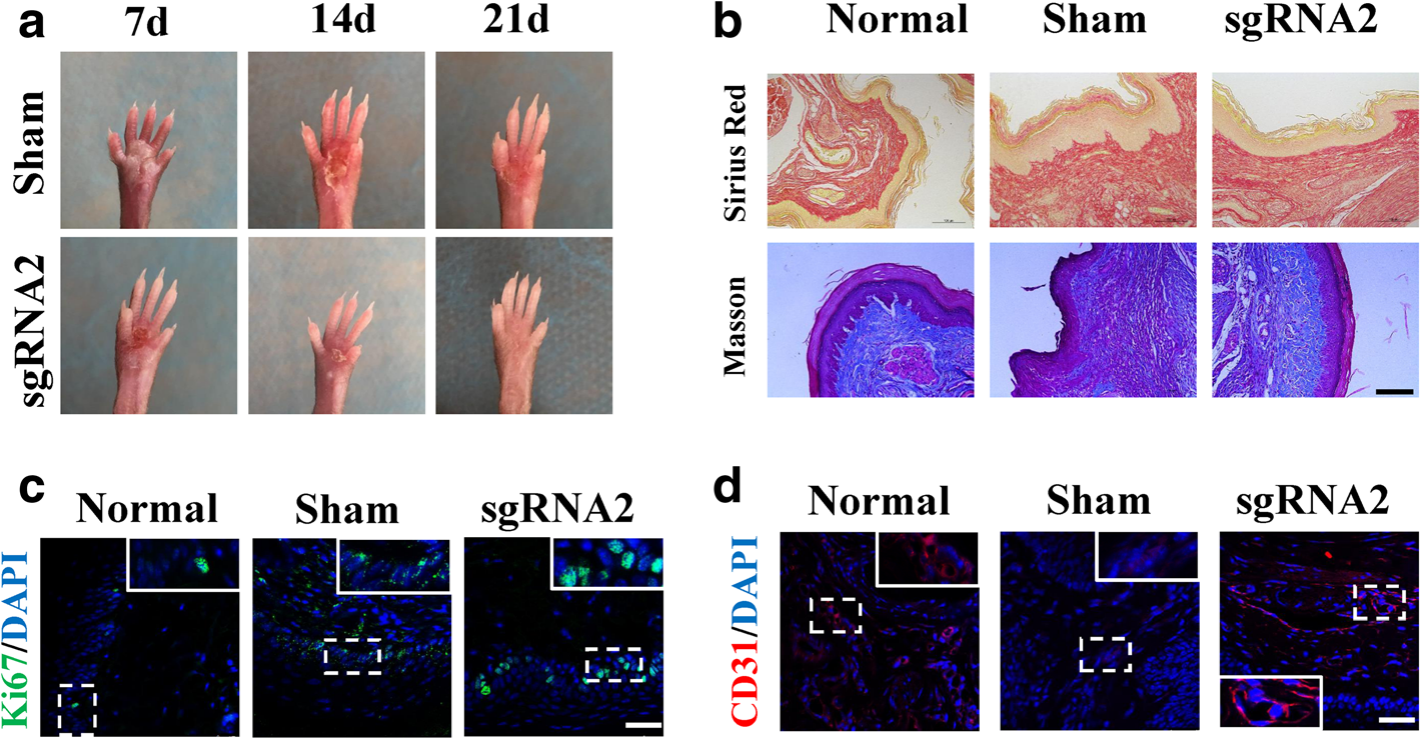
The effect of Dox-induced dCas9-E BM-MSCs on wound healing in vivo. **a** The mice were treated with Dox-induced dCas9-E BM-MSCs after full thickness scald injuries. **b** Sirius Red staining and Masson staining were used to detect the fibrosis, and the arrangement of collagen fibers was more regular than in the sham-treated group. Immunostaining was performed for **c** Ki67 (green) and **d** CD31 (red) after treatment with Dox-induced dCas9-E BM-MSCs. Scale bars = 20 μm. DAPI 4’6-diamidino-2-phenylindole, sgRNA single-guide RNA

**Fig. 6:**
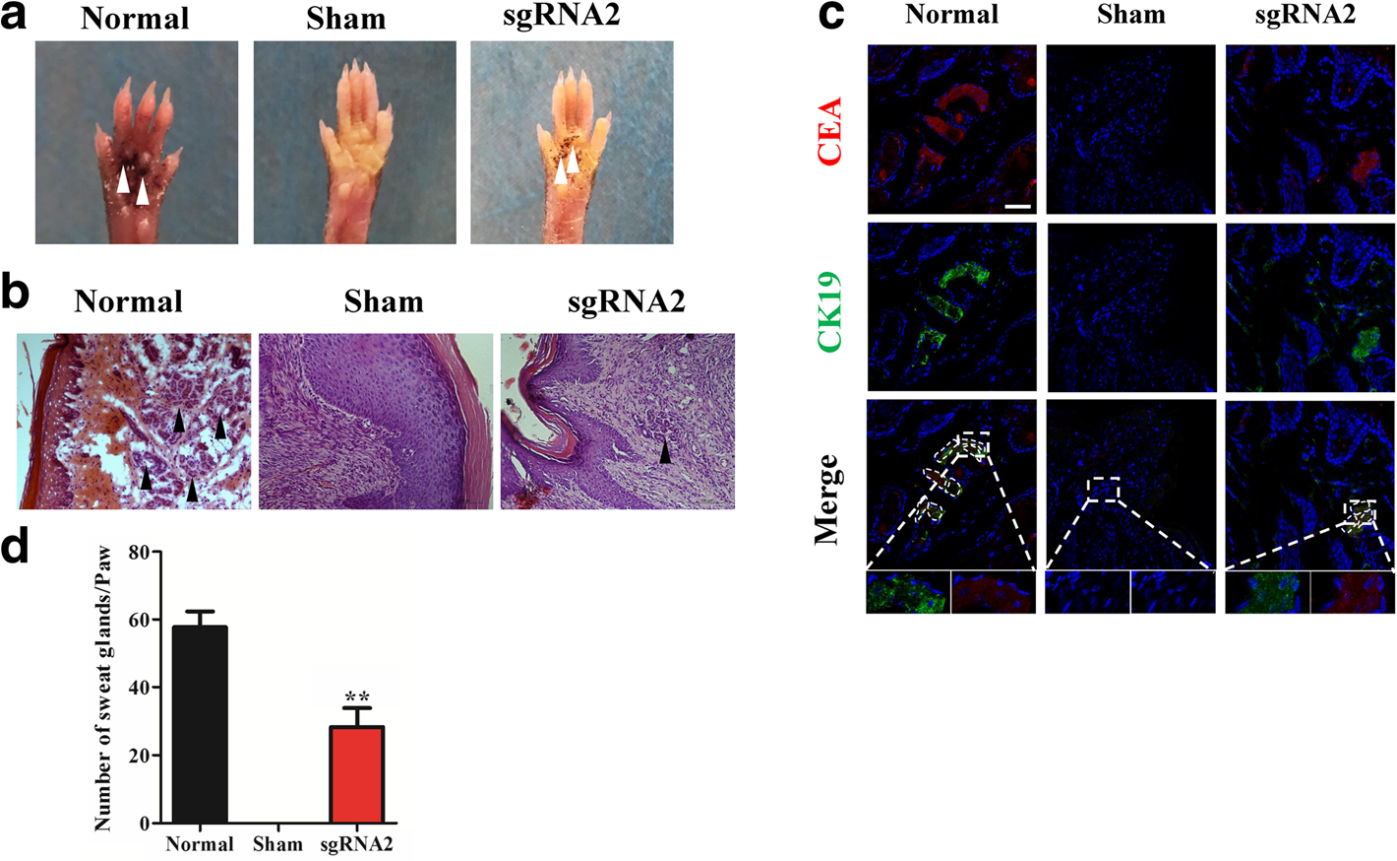
The effect of dCas9-E BM-MSCs on the regeneration of sweat glands in vivo. **a** The saline-treated paw (Sham) showed a negative result for the perspiration test, whereas paws implanted with Dox-induced dCas9-E BM-MSCs showed a positive result (sgRNA2). **b** Hematoxylin and eosin staining was used to detect the duct structure of sweat gland tissue after injection with Dox-induced dCas9-E BM-MSCs. **c** The expression of carcinoembryonic antigen (CEA; red), cytokeratin 19 (CK19; green) and nucleus (DAPI) in BM-MSCs after implantation for 20 days. **d** The quantification of each paw (0.5 cm × 0.5 cm, treated with collagenase I for 1 h at 37 °C) was also conducted after treatment with Dox-induced dCas9-E BM-MSCs. **p < 0.01 for Dox-induced dCas9-E BM-MSCs group versus normal group. Scale bar = 50 μm. sgRNA single-guide RNA

A sweat gland injured mouse model was used to examine the effect of dCas9-E BM-MSCs on repair and regeneration of injured tissue. Samples of the injured area were collected every week to evaluate the speed of wound healing. The experimental group treated with Dox-induced dCas9-E BM-MSCs showed a faster healing speed (Fig. 5a), and hematoxylin and eosin staining also demonstrated accelerated re-epithelialization in the experimental group (Additional file 3), with less collagen deposition than in the sham group (Fig. 5b). Immunofluorescence of Ki-67 and CD31 confirmed that dCas9-E BM-MSCs induced by Dox had more proliferation of cells and generated microvascular tissue, resulting in accelerated scarless wound healing (Fig. 5c, d). These results demonstrated that the Dox-induced dCas9-E BM-MSCs can promote wound healing with less fibrosis after injury. At 20 days after treatment with Dox-induced dCas9-E BM-MSCs, eight of ten treated paws were positive for the iodine-starch perspiration test with a distinctive blue and black area. However, the sham-treated paws showed a negative result compared with normal paw skin (Fig. 6a). Hematoxylin and eosin staining showed the duct structure of sweat gland tissue in the experimental and control groups (Fig. 6b). The tissue immunofluorescence from the experimental and control paws indicated that the paw skin treated with Dox-induced dCas9-E BM-MSCs could exhibit the SG biomarkers (Fig. 6c). The number of sweat gland tissues in the experiment group treated with Dox-induced dCas9-E BM-MSCs were significantly higher compared with the sham group (Fig. 6d). These results indicated that Dox-induced dCas9-E BM-MSCs could act as SGCs and promote wound healing.

### Tumorigenicity test in animals

**Fig. 7:**
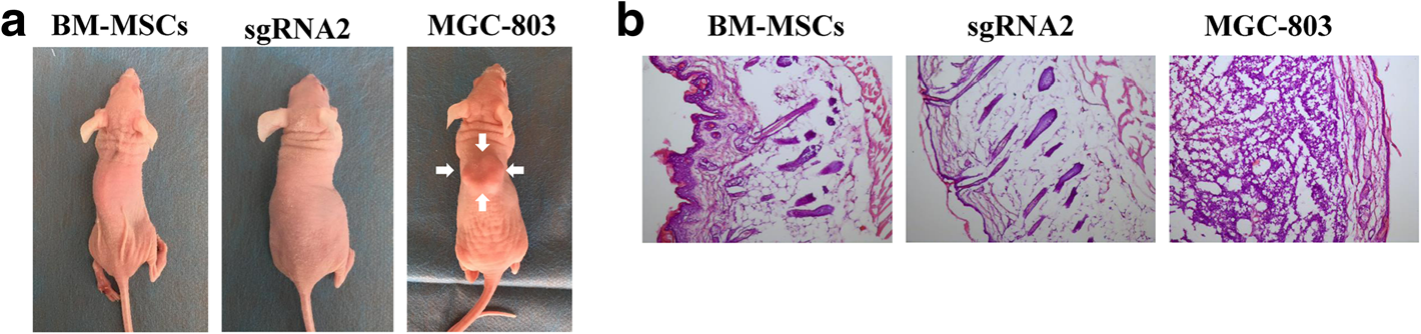
Rule-out tumorigenicity assay. Athymic BALB/c nude mice were injected subcutaneously with 5 × 10^5^ Dox-induced dCas9-E bone marrow-derived mesenchymal stem cells (BM-MSCs) suspended in DMEM (FBS free). Positive control mice were injected with MGC-803 cells and sent to pathology analysis when tumors were ≤ 1.0 cm in diameter. Representative images (**a**) and hematoxylin and eosin staining (**b**) showed tumor formation after cell injection. sgRNA single-guide RNA

The tumorigenicity test for Dox-induced dCas9-E BM-MSCs in mice was conducted at a specific pathogen-free (SPF) facility for 12 weeks. Mice were inspected for tumor development every 3 days for 12 weeks after injection of the indicated cells. No evidence of tumor formation was found in any of the 10 mice treated with Dox-induced dCas9-E BM-MSCs, while 10 out of 10 mice injected with positive control cells (MGC803 cells) grew tumors within 1 month after cell injection (Fig. 7). The results indicated that the induced SGCs did not induce tumors at doses up to 5 × 10^5^ cells.

### Enhanced expression of Shh and cyclin D1 by activation of EDA

**Fig. 8:**
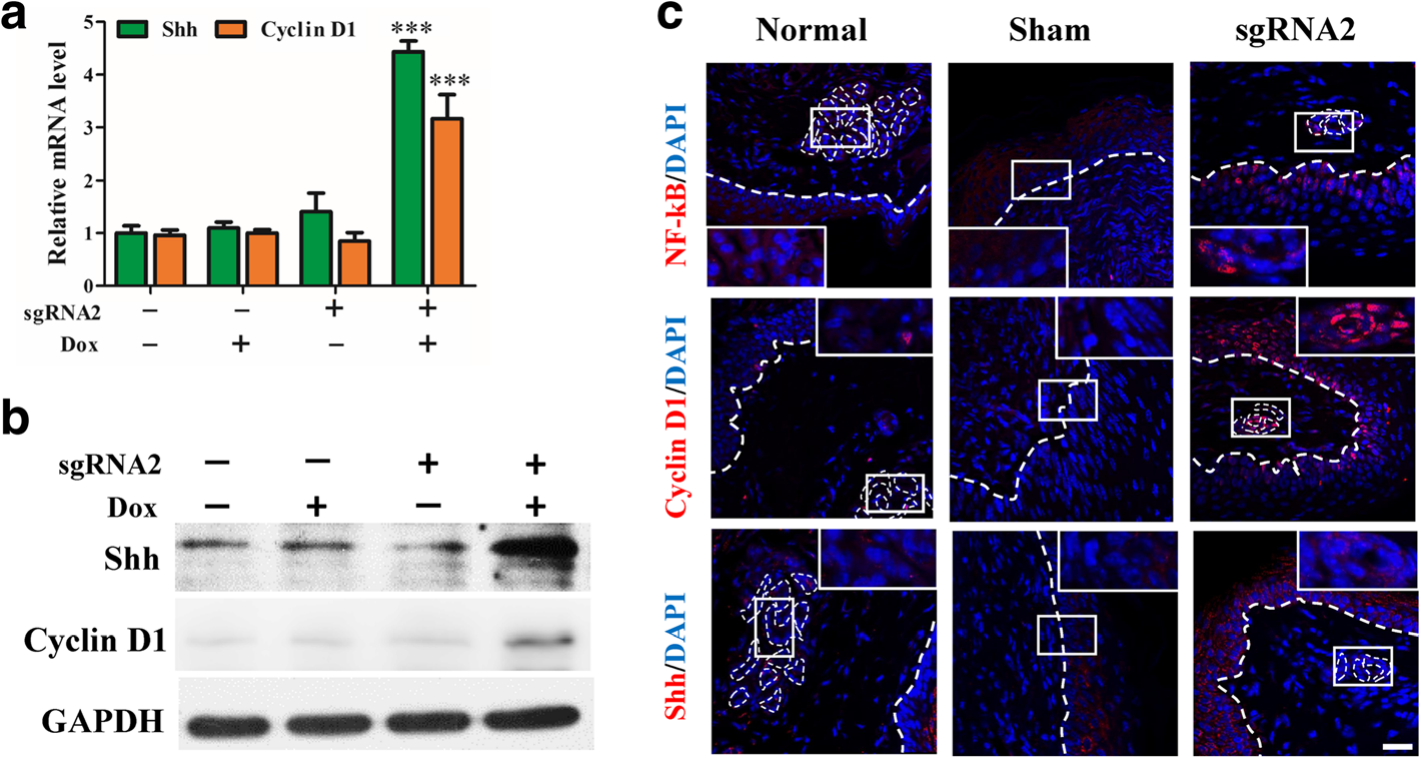
Involvement of NF-κB pathways in dCas9-E-induced EDA expression. The cells transfected with dCas9-E and incubated with doxycycline (Dox; 2 μg/ml) for 48 h exhibited a significance difference in NF-κB downstream Sonic Hedgehog (Shh) and cyclin D1 mRNA (**a**) and protein (**b**) expression. Immunostaining was also performed to detect the activity of the NF-κB pathway. dCas9-E BM-MSCs induced more NF-κB translocated into the nuclei and then activated the expression of Shh and cyclin D1 (**c**). All data are normalized to glyceraldehyde-3-phosphate dehydrogenase (GAPDH) and calibrated based on the BM-MSC group. ***p <0.001 for Dox-treated dCas9-E BM-MSCs versus BM-MSCs. Scale bar = 20 μm. DAPI 4’6-diamidino-2-phenylindole, sgRNA single-guide RNA

The EDA/EDAR pathway could activate NF-κB through the IKK pathway. Shh and cyclin D1, the downstream genes of NF-κB, were detected by qRT-PCR (Fig. 8a) and Western blotting (Fig. 8b). Since dCas9-E is a Dox-inducible protein, the dCas9-E BM-MSCs treated with Dox could express higher levels of Shh and cyclin D1 than those which were not treated with Dox (Fig. 8a, b). Immunofluorescence showed that NF-kB activation increased cyclin D1 and Shh expression in the epidermis and newly generated sweat glands in the experimental group (Fig. 8c). These results indicated that dCas9-E BM-MSCs treated with Dox could activate the NF-κB pathway, enhancing the expression of Shh and cyclin D1.

## Discussion

Clinical treatment for, and research into, the structural and functional repair and regeneration of cutaneous tissue after severe burn injury is a huge challenge. Recently, cell therapy has emerged as a new tool to repair the injured skin structure and re-establish the sweating function [1, 3, 6, 15].

With easy accession and culture, a strong differentiation potency, and lower immune resistance, BM-MSCs are considered to be promising cells for cutaneous regeneration in severe burn patients. In the present study, we have demonstrated that direct activation of the EDA gene has great potential for facilitating the formation of sweat gland-like cells via the doxycycline-induced dCas9-E system, giving us a chance to improve the current methods of sweat gland cell generation.

Previous studies have demonstrated that protein encoded by the *EDA* gene could regulate the development of ectodermal tissues such as sweat glands, hair, and teeth [20]. People who lack the *EDA* gene have sparse or absent hair and teeth. These results and phenomenon indicate that EDA acts as a key regulator in the initiation stage of cutaneous appendage development [18, 21]. Thus, we hypothesized that activation of the *EDA* gene could facilitate BM-MSC differentiation into sweat glands.

The sgRNA-guided dCas9-E system is a newly developed system to activate transcription of the target gene [7, 22]. In the present study, three different sgRNAs were designed to target the –244 bp to –58 bp region upstream of the TSS in the human EDA promoter. Interestingly, individual sgRNAs targeting the EDA promotor around the –244 bp to –58 bp region could effectively increase the transcriptional activation in the presence of dCas9-E (Fig. 3), and the sgRNA2 around the –244 bp to –225 bp region showed higher activity than sgRNA1 (from –131 bp to –112 bp) and sgRNA3 (from –78 bp to –59 bp), suggesting that target position is involved in the activity of the dCas9-E.

Sweat gland epithelial cells have been identified as essential for the construction of skin substitutes and regeneration of sweat glands [23]. Several markers, including CEA, CK7, CK8, CK14, CK15, CK18, and CK19, were identified as the sweat gland-specific markers during SG development. In normal adult skin tissue, these markers (CK7, CK8, CK14, CK15, CK18, and CK19) are expressed in the secretory portion [5, 24]. In addition, CEA, a biomarker for colorectal cancer, is also expressed in some normal adult tissue, such as mucous neck cells and pyloric mucous cells in the stomach, and secretory epithelia and duct cells of sweat glands [25]. Therefore, the high expression of CEA, CK7, CK8, CK14, CK15, CK18, and CK19 is considered as an index for the identification of SGs [15, 23, 26]. After transfection with sgRNA-guided dCas9-E, the BM-MSCs were identified as showing high expression of CEA, CK7, CK14, and CK19 (Fig. 4). These results indicated that activation of the EDA gene could induce BM-MSCs into sweat gland-like cells. The function of the transfected BM-MSCs was measured between treated paws and sham paws in vivo. Results showed that paws treated with Dox-induced dCas9-E BM-MSCs had accelerated wound healing with less collagen deposition (Fig. 5a, b). Primary research has shown that the wound healing process depends on cell proliferation in the basement membrane of the wound edge [27]. Immunofluorescence staining of Ki67 in the Dox-induced dCas9-E BM-MSC treatment group showed higher proliferation in the basement membrane cells compared with the sham group after newly complete re-epithelialization (Fig. 5c), while the number of Ki67-positive cells decreased in the stabilized healing site (Additional file 4), in accordance with changes in MSC-based wound healing therapy [28]. Therefore, we concluded that the transfected cells showed partly preserved MSC features. In addition, hematoxylin and eosin staining and immunofluorescence staining also indicated that the scalded paws treated with Dox-induced dCas9-E BM-MSCs showed sweat gland duct structure and positive markers (CEA and CK19) for sweat glands (Fig. 6). However, the mechanism for EDA regulation of sweat gland development was still unclear.

Previous studies have revealed that multiple signaling pathways, such as ERK-MAPK, EDA/EDAR/NF-κB, and Wnt/β-catenin, were involved in sweat gland development [29, 30]. EDA, a member of the TNF superfamily, is one of the functional genes that regulate the development of sweat glands, and mutations in EDA can cause ectodermal dysplasia in humans and lead to sweat-free syndrome [31]. In the present study, therefore, we focused on the EDA/EDAR/NF-κB pathway. The EDA pathway could activate NF-κB through the IKK pathway, and the activated NF-κB can enter the nucleus to promote the expression of cyclin D1, Shh, Fox family genes, and keratins [15], which play an important role in the development of sweat glands [32]. We further found that the EDA/EDAR/NF-κB signaling pathway was activated in the reprogrammed sweat gland-like cells and the injured site in vivo. The activated NF-κB then activates the expression of Shh and cyclin D1 downstream (Fig. 8). In combination with the in vivo experimental results, we have shown a new therapeutic direction in sweat gland regeneration by revealing a promising technology for direct and effective reprogramming.

## Conclusions

In conclusion, our findings demonstrated that induction of EDA gene overexpression via dCas9-E could promote BM-MSCs to transform into sweat gland-like cells, thereby generating a therapeutic potential for patients with destroyed sweat glands and extensive deep burns. The sgRNAs targeting the EDA promotor and dCas9-E were transferred into BM-MSCs and exhibited a sweat gland-like phenotype. Moreover, our studies with an in vivo SG-injured mouse model showed that the therapeutic effect of dCas9-E BM-MSCs on SG injuries was enhanced by induction of Dox. Taken together, our data show that targeting the EDA promotor by dCas9-E could effectively induce the reprogramming of BM-MSCs into sweat gland-like cells.

## Additional files

Additional file 1: Primer sequence information for RT-qPCR amplification. (XLSX 11 kb)

Additional file 2: Identification of BM-MSCs. Flow cytometry analysis was used to identify phenotypes of BM-MSCs. The cells were labeled with the following biomarkers: FITC-conjugated anti-HLA-DR, anti-CD34, anti-CD45, anti-CD90, and PE-conjugated anti-CD73, and anti-CD90. BM-MSCs were positive for all three MSCs biomarkers, but negative for hematological, pan-leukocyte and HLA-DR markers (A). Multipotent differentiation capacity of BM-MSCs. BM-MSCs were cultured in osteogenic and adipogenic induction medium for 4 weeks. The differentiated cells were positive by specific methods: alkaline phosphatase for osteogenic cells (B) and Oil Red O for adipogenic cells (C). Scale bars = 50 μm. (PPTX 1177 kb)

Additional file 3: Hematoxylin and eosin staining for paw re-epithelialization after scald injury. The paws of each mice treated with or without Dox-induced dCas9-E BM-MSCs were collected after scald injury for 7 to 21 days. The complete healing epithelial layer is labeled with a dotted line. (PPTX 2327 kb)

Additional file 4: Immunofluorescence staining of Ki67 for stabilized re-epithelialization site. (PPTX 4167 kb)

## Abbreviations

BM-MSC: Bone marrow-derived mesenchymal stem cell
BSA: Bovine serum albumin
Cas: CRISPR-associated system
CEA: Carcinoembryonic antigen
CK: Cytokeratin
CRISPR: Clustered Regularly Interspaced Short Palindromic Repeats
DAPI: 4’6-Diamidino-2-phenylindole
dCas9-E: dCas9-effector
DMEM: Dulbecco’s modified Eagle’s medium
Dox: Doxycycline
EDA: Ectodysplasin
EDAR: EDA receptor
EGF: Epidermal growth factor
FBS: Fetal bovine serum
FGF: Fibroblast growth factor
FITC: Fluorescein isothiocyanate
GAPDH: Glyceraldehyde-3-phosphate dehydrogenase
HA: Hemagglutinin
PE: Phycoerythrin
PVDF: Polyvinylidene difluoride
qRT-PCR: Quantitative reverse transcription polymerase chain reaction
SG: Sweat gland
SGC: Sweat gland-like cell
sgRNA: Single-guide RNA
Shh: Sonic Hedgehog
TEM: Transmission electron microscopy
TNF: Tumor necrosis factor
TRE: Tetracycline-responsive element
TSS: Transcriptional start site
