# Supplementary material for: Targeting ectodysplasin promotor by CRISPR/dCas9-effector effectively induces the reprogramming of human bone marrow-derived mesenchymal stem cells into sweat gland-like cells
Source: Stem Cell Res Ther. 2018 Jan 12;9:8. doi: 10.1186/s13287-017-0758-0 (PMC5766979; doi:10.1186/s13287-017-0758-0)
Supplement: Supplementary file 2 — Identification of BM-MSCs. Flow cytometry analysis was used to identify phenotypes of BM-MSCs. The cells were labeled with the following biomarkers: FITC-conjugated anti-HLA-DR, anti-CD34, anti-CD45, anti-CD90, and PE-conjugated anti-CD73, and anti-CD90. BM-MSCs were positive for all three MSCs biomarkers, but negative for hematological, pan-leukocyte and HLA-DR markers (A). Multipotent differentiation capacity of BM-MSCs. BM-MSCs were cultured in osteogenic and adipogenic induction medium for 4 weeks. The differentiated cells were positive by specific methods: alkaline phosphatase for osteogenic cells (B) and Oil Red O for adipogenic cells (C). Scale bars = 50 μm. (PPTX 1177 kb) [file 13287_2017_758_MOESM2_ESM.pptx]

## Slide 1
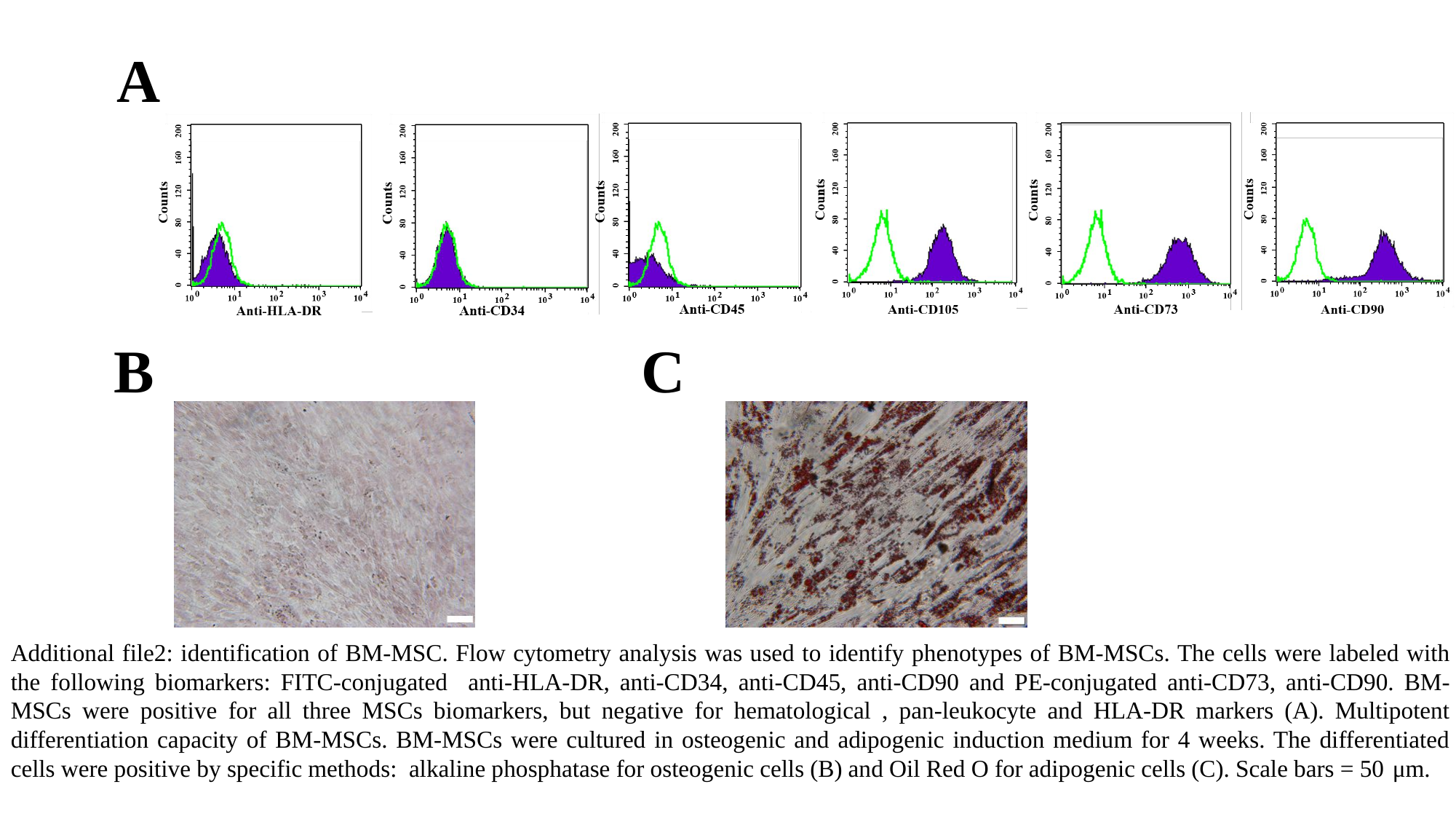

A
B
C
Additional file2: identification of BM-MSC. Flow cytometry analysis was used to identify phenotypes of BM-MSCs. The cells were labeled with the following biomarkers: FITC-conjugated anti-HLA-DR, anti-CD34, anti-CD45, anti-CD90 and PE-conjugated anti-CD73, anti-CD90. BM-MSCs were positive for all three MSCs biomarkers, but negative for hematological , pan-leukocyte and HLA-DR markers (A). Multipotent differentiation capacity of BM-MSCs. BM-MSCs were cultured in osteogenic and adipogenic induction medium for 4 weeks. The differentiated cells were positive by specific methods: alkaline phosphatase for osteogenic cells (B) and Oil Red O for adipogenic cells (C). Scale bars = 50 μm.
