# Supplementary material for: Targeting ectodysplasin promotor by CRISPR/dCas9-effector effectively induces the reprogramming of human bone marrow-derived mesenchymal stem cells into sweat gland-like cells
Source: Stem Cell Res Ther. 2018 Jan 12;9:8. doi: 10.1186/s13287-017-0758-0 (PMC5766979; doi:10.1186/s13287-017-0758-0)
Supplement: Supplementary file 3 — Hematoxylin and eosin staining for paw re-epithelialization after scald injury. The paws of each mice treated with or without Dox-induced dCas9-E BM-MSCs were collected after scald injury for 7 to 21 days. The complete healing epithelial layer is labeled with a dotted line. (PPTX 2327 kb) [file 13287_2017_758_MOESM3_ESM.pptx]

## Slide 1
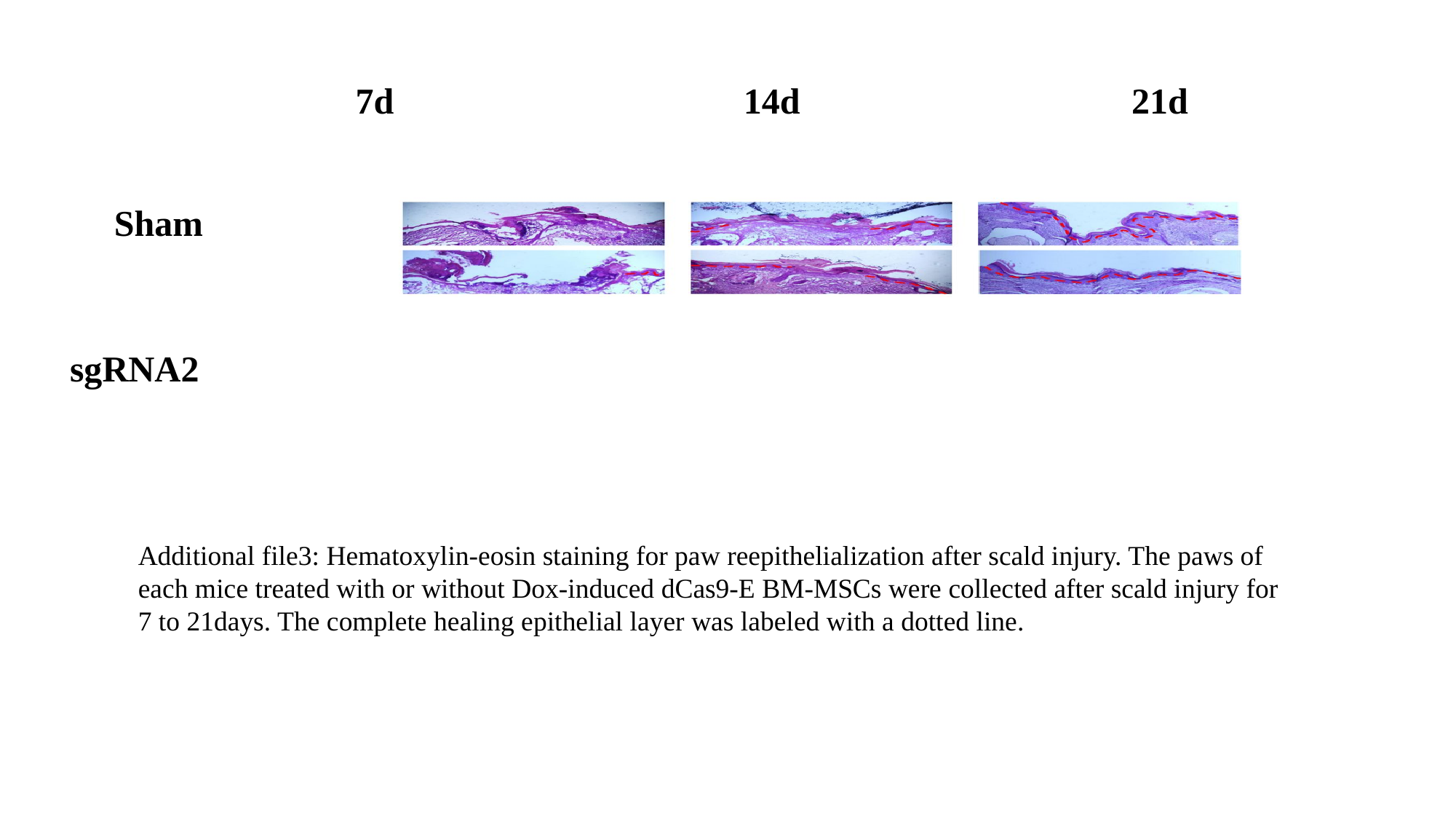

7d
14d
21d
Sham
sgRNA2
Additional file3: Hematoxylin-eosin staining for paw reepithelialization after scald injury. The paws of each mice treated with or without Dox-induced dCas9-E BM-MSCs were collected after scald injury for 7 to 21days. The complete healing epithelial layer was labeled with a dotted line.
