# Supplementary material for: Targeting ectodysplasin promotor by CRISPR/dCas9-effector effectively induces the reprogramming of human bone marrow-derived mesenchymal stem cells into sweat gland-like cells
Source: Stem Cell Res Ther. 2018 Jan 12;9:8. doi: 10.1186/s13287-017-0758-0 (PMC5766979; doi:10.1186/s13287-017-0758-0)
Supplement: Supplementary file 4 — Immunofluorescence staining of Ki67 for stabilized re-epithelialization site. (PPTX 4167 kb) [file 13287_2017_758_MOESM4_ESM.pptx]

## Slide 1
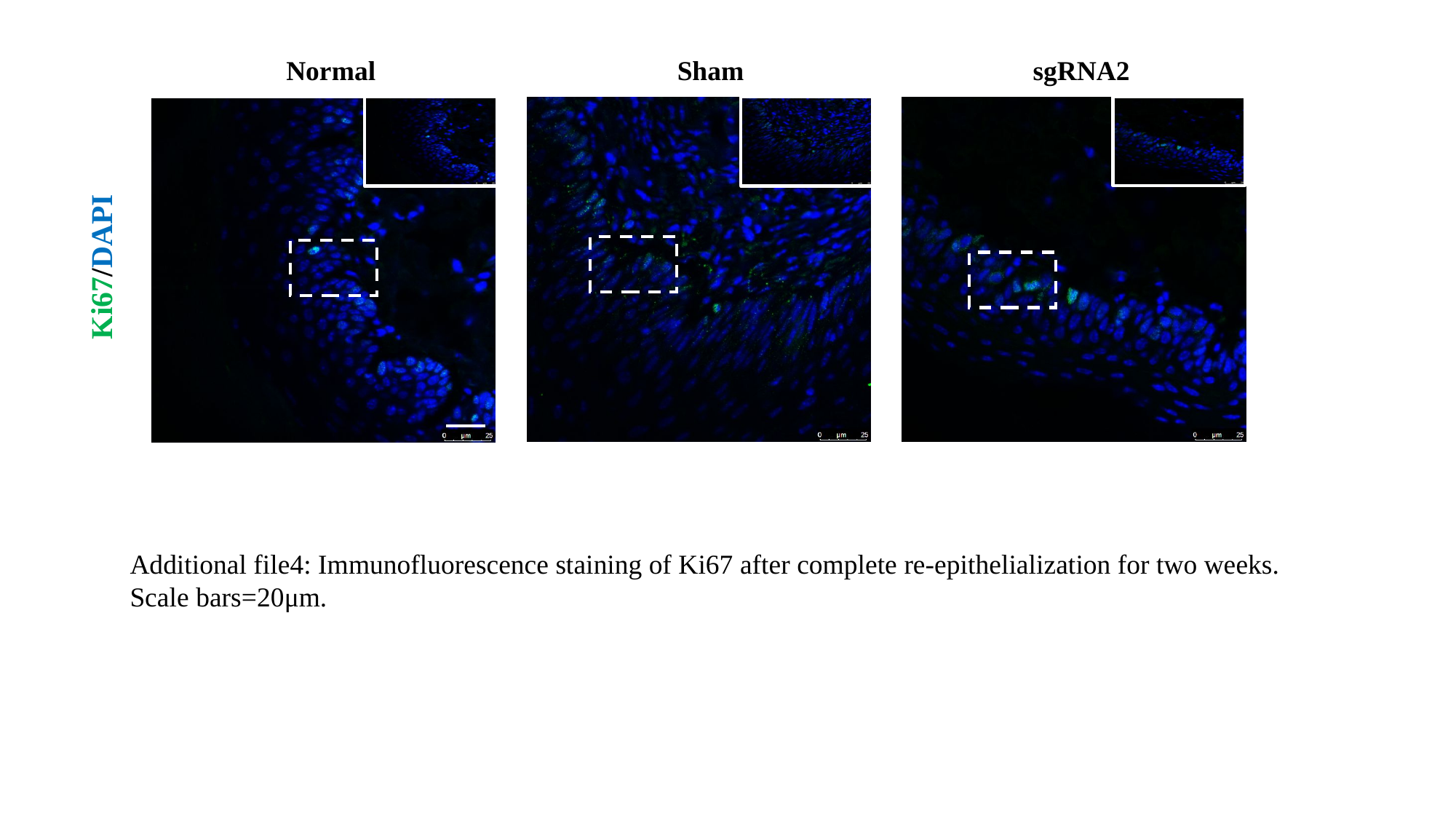

Normal
Sham
sgRNA2
Ki67/DAPI
Additional file4: Immunofluorescence staining of Ki67 after complete re-epithelialization for two weeks. Scale bars=20μm.
